# The relationship between inflammation and neurocognitive dysfunction in obstructive sleep apnea syndrome

**DOI:** 10.1186/s12974-020-01905-2

**Published:** 2020-08-01

**Authors:** Xiangming Liu, Yiming Ma, Ruoyun Ouyang, Zihang Zeng, Zijie Zhan, Huanhuan Lu, Yanan Cui, Zhongshang Dai, Lijuan Luo, Chenjie He, Herui Li, Dandan Zong, Yan Chen

**Affiliations:** 1 Department of Respiratory and Critical Care Medicine, the Second Xiangya Hospital, Central South University, Changsha, 410011 Hunan China; 2 Research Unit of Respiratory Disease, Central South University, Changsha, 410011 Hunan China

**Keywords:** OSAS, Microglia activation, Inflammation, Neuroinflammation, Neurocognitive dysfunction, Animal model

## Abstract

Obstructive sleep apnea syndrome (OSAS), a state of sleep disorder, is characterized by repetitive apnea, chronic hypoxia, oxygen desaturation, and hypercapnia. Previous studies have revealed that intermittent hypoxia (IH) conditions in OSAS patients elicited neuron injury (especially in the hippocampus and cortex), leading to cognitive dysfunction, a significant and extraordinary complication of OSAS patients. The repeated courses of airway collapse and obstruction in OSAS patients resulted in apnea and arousal during sleep, leading to IH and excessive daytime sleepiness (EDS) and subsequently contributing to the development of inflammation. IH-mediated inflammation could further trigger various types of cognitive dysfunction. Many researchers have found that, besides continuous positive airway pressure (CPAP) treatment and surgery, anti-inflammatory substances might alleviate IH-induced neurocognitive dysfunction. Clarifying the role of inflammation in IH-mediated cognitive impairment is crucial for potentially valuable therapies and future research in the related domain. The objective of this article was to critically review the relationship between inflammation and cognitive deficits in OSAS.

## Introduction

Obstructive sleep apnea syndrome (OSAS), characterized by recurrent courses of complete or partial collapse of the upper airway during sleep, is a state of sleep disorder that has become a significant public health problem over time. The features of OSAS include snoring, sleep fragmentations, and excessive daytime sleepiness (EDS). Additionally, the collapse of the upper airway directly leads to repetitive apnea, chronic hypoxia, oxygen desaturation, and hypercapnia [1]. Obesity, age, and gender appear to be three of the most important risk factors associated with OSAS [2]. Due to the advances in diagnostic equipment and evolvement of diagnostic criteria, the prevalence of OSAS in the population has increased with time [3]. The adverse medical conditions associated with OSAS include cardiovascular disease [4–6] (coronary artery disease, heart failure, atrial fibrillation, hypertension, and stroke), metabolic dysfunction [7, 8] (dyslipidemia and diabetes mellitus), and neurocognitive impairment [9, 10]. Recently, cognitive impairment has been proven to be an important complication of OSAS and deserves enough attention. A broad domain of cognitive functions, including memory, attention/vigilance, and executive function, is involved in this process [9]. Many studies have proven that systemic and neuroinflammation play a crucial role in neurocognitive dysfunction [11–13]. In this article, recent papers concerning the relationship between inflammation and cognitive impairment in OSAS will be summarized to further help researchers to understand this problem more clearly.

## Inflammation in OSAS

The main mechanisms of OSAS are hypoxia and oxidative stress; however, several studies have demonstrated that inflammation also plays a crucial role in the occurrence and development of OSAS [14]. Systemic and local inflammation in OSAS patients manifests as a result of chronic intermittent hypoxemia (CIH) [11, 15], snoring [16], continuous hypoxia [17], oxidative stress, and sleep fragmentation and deprivation [18–20]. In the last two decades, many studies have investigated the precise interactions between OSAS and inflammation in both adults and children, and some studies have tried to elucidate the potential biomarkers in the presence of OSAS and OSAS-related morbidities [21–23].

### Biomarkers of Inflammation

Data provided by numerous studies have proven that inflammation induced by OSAS might trigger the impairment of the vascular endothelial cells and further modify the structure and function of vessels [24]. This impairment leads to endothelial dysfunction, which contributes to various end-organ morbidities, such as cardiovascular disease, metabolic dysfunction and more importantly, the impairment of neurocognitive function [25, 26]. Serum biomarkers related to inflammation, such as interleukin (IL)-1, IL-6, IL-8, IL-17, IL-23, IL-33, tumor necrosis factor-α (TNF-α), nuclear factor kappa B (NF-κB), interferon-γ (IFN-γ), high mobility group box 1 (HMGB1), high-sensitivity C-reactive protein (hs-CRP), serum amyloid A (SAA), prostaglandin E2 (PGE2), uric acid (UA), nitric oxide (NO), P2X7 receptor (P2X7R), toll-like receptors (TLRs), receptor for advanced glycation end product (RAGE), peroxisome proliferators-activated receptor γ (PPAR-γ), intercellular adhesion molecules (ICAM), vascular cell adhesion molecule (VCAM), vascular endothelial growth factor (VEGF), pentraxin-3 (PTX-3), leptin, selectins fibrinogen, NLRP3 inflammasome, myeloid-related protein 8/14 (MRP 8/14), monocyte chemoattractant protein-1 (MCP-1), macrophage migration inhibitory factor (MIF), CC motif chemokine ligand 5 (CCL5), cyclooxygenase-2 (COX-2), and inducible nitric oxide synthase (iNOS) are activated in OSAS patients [27–32].

Many studies have suggested that OSAS can result in systemic and local inflammation in OSAS patients (Table 1). A meta-analysis focusing on OSAS and inflammation noted that compared with the control group, OSAS patients presented significantly elevated CRP, TNF-α, IL-6, IL-8, ICAM, VCAM, and selectins levels. They found the most prominent inflammatory factors presented in OSAS include IL-1, IL-6, and CRP. Additionally, they found that alterations in cytokine levels were closely correlated with the age, body mass index (BMI), and apnea-hypopnea index (AHI) of the patients [33]. A single-center, cross-sectional study conducted by Bouloukaki and colleagues included 1053 subjects who were free of comorbidities and were grouped according to AHI into control, mild, moderate, and severe groups. They collected venous blood from all subjects to measure the levels of CRP, fibrinogen, erythrocyte sedimentation rate (ESR), and UA. The levels of hs-CRP and fibrinogen were elevated significantly in the severe group compared with those in the mild group. However, no significant difference was found between the mild and moderate groups; the UA levels were significantly different among the other groups. However, no obvious difference was found among the groups for ESR. Interestingly, all of these biomarkers except fibrinogen were correlated with sleep time spent with SaO_2_ less than 90% (TST90), and fibrinogen was related to active smoking [34]. Similar studies conducted by other researchers demonstrated parallel results; CRP was positively correlated with AHI, even adjusted for BMI [39–42].

**Table 1:** Biomarkers of inflammation in OSAS patients

Reference	Number of subjects	Detecting parameter	Cytokines levels alteration	Cytokines concentrations correlated with
Nadeem et al. 2013 [33]	Meta-analysis of 2952 OSAS and 2784 controls	CRP, TNF-α, IL-6, IL-8, ICAM, VCAM, and selectins	CRP↑, TNF-α↑, IL-6↑, IL-8↑, ICAM↑, VCAM↑, and selectins↑	Age, BMI, AHI
Bouloukaki et al. 2017 [34]	858 OSAS; 190 controls	CRP, fibrinogen, UA and ESR	CRP↑, fibrinogen↑, UA↑	Gender, BMI, AHI, ODI and mean and minimum SaO_2_, TST90 and active somking
Bozic et al. 2018 [35]	50 OSAS; 25 controls	TNF-α, IL-6 and hsCRP	TNF-α↑, IL-6↑, hsCRP↑	Plasma adropin levels
Motamedi et al. 2018 [36]	50 OSAS; 24 controls	Tau, IL-6, IL-10, CRP, TNF-α and Aβ	Tau↑, IL-6↑	AHI
Svatikova et al. 2003 [37]	20 OSAS; 17 controls	SAA	SAA↑	AHI
Sozer et al. 2018 [38]	60 OSAS; 24 controls	CRP, PTX-3, ProCT, IL-33 and sST2	PTX-3↑, IL-33↑, and sST2↑	BMI, ODI, minimum SaO_2_

*CRP* C-reactive protein, *TNF*-*α* tumor necrosis factor-α, *IL* interleukin, *ICAM* intercellular adhesion molecules, *VCAM* vascular cell adhesion molecule, *BMI* body mass index, *AHI* apnea hypopnea index, *UA* uric acid, *ESR* erythrocyte sedimentation rate, *ODI* oxygen desaturation index, *TST90* sleep time spent with SaO_2_ less than 90%, *Aβ* amyloid beta, *SAA* serum amyloid A, *PTX*-*3* pentraxin-3, *ProCT* procalcitonin, *sST2* soluble receptor ST2

TNF-α is involved in the regulation of sleep and promotes nonrapid eye movement sleep, and the concentration of TNF-α in human body exhibits circadian patterns [43]. The TNF-α levels increase after undergoing sleep fragmentation and deprivation [22, 44–46]. Bozic et al. [35] screened 50 subjects with newly diagnosed OSAS (25 moderate and 25 severe OSAS patients) and 25 healthy controls. The results showed that the TNF-α, IL-6, and hsCRP levels in the severe group were significantly higher than those in the moderate and healthy control groups. Additionally, the subjects harboring the TNF-α-308G gene polymorphism tended to exhibit symptoms of daytime sleepiness [47]. Similar results also appeared in obese populations [48].

IL-6 has recently received considerable attention due to its ability to cause vascular inflammation and promote cardiovascular disease, diabetes, and cognitive function deterioration [49–51]. Tau, a microtubule-related protein, is essential for normal neuronal activity and, together with amyloid beta (Aβ), is associated with neurodegenerative processes and neuronal death [52]. CIH would increase total tau level by upregulating tau phosphorylation in OSAS [26, 53]. Motamedi et al. [36] assessed the levels of tau, IL-6, IL-10, CRP, and TNF-α in three groups (24 healthy control subjects, 22 mild, and 28 moderate-severe OSAS subjects). Compared with the control and mild OSAS groups, the tau and IL-6 concentrations were significantly increased in the severe group. This difference remained significant after adjusting for potential confounding factors such as age, race, gender, and BMI. Additionally, they found that elevated tau levels were associated with AHI. However, no significant difference was found in the TNF-α, IL-10, and CRP levels among the three groups. This discrepancy may be related to the small sample size of the survey, younger subject population, and mostly male gender of the subjects.

Furthermore, Svatikova and colleagues performed a study to observe the major acute-phase protein SAA in OSAS patients [37]. They found the SAA levels in moderate to severe OSAS were 2.5-fold higher than those in healthy and mild OSAS subjects. The concentrations of PTX-3, procalcitonin (ProCT), CRP, IL-33, and its soluble receptor ST2 (sST2) were measured to observe their importance as biomarkers in OSAS. Higher levels of PTX-3, IL-33, and ST2 were observed in the OSAS groups than the control group while other cytokines such as ProCT and CRP exhibited similar levels in all groups. Moreover, their data suggested that PTX-3, as an inflammatory factor, may play an important role as an indicator of OSAS severity [38].

### Inflammation Levels after CPAP treatment

CPAP is generally considered a first-line treatment for patients with OSAS. Early use of CPAP can provide patients with maximum functional recovery and minimal residual symptoms [54–58]. Surgery, as a treatment for OSAS patients, is not an alternative to CPAP but a rescue measure after the failure of CPAP or other conservative therapies [59].

Several studies have manifested a significant decline in the level of inflammation in OSAS patients after CPAP treatment (Table 2). A recent meta-analysis concluded that CPAP therapy could significantly decrease the levels of CRP, IL-6, IL-8, and TNF-α in OSAS patients. A longer therapy duration (> 3 months) and adequate compliance (≥ 4 h/night) were also found to more effectively promote a decrease in systemic inflammation [69]. In a large population of CPAP-treated OSAS patients, Schiza et al. [60] assessed the CRP levels for the 12-month follow-up period and found that the concentration of CRP showed a gradual decrease at 3 months with a steep decline at 6 months, reaching a plateau thereafter. They also divided the patients into good and poor compliance groups with CPAP therapy, and the CRP evolution pattern mentioned above was only found in the good compliance group. Relevant conclusions were also reached by Steiropoulos et al. [61] who recruited 52 newly diagnosed OSAS patients and treated them with CPAP. After 6 months of treatment, the patients were divided into 2 groups: good compliance group (mean CPAP use ≥ 4 h/night) and poor compliance group (mean CPAP use < 4 h/night). The serum levels of TNF-α and UA, along with CD4^+^ cell counts, were only decreased in the former group. These data suggest that the inflammatory levels of the body can only be improved after an adequate CPAP treatment time. Yokoe et al. [62] assessed the concentration of CRP and IL-6 in two groups (30 newly diagnosed OSAS patients and 14 male obese control subjects) and found that the levels of CRP and IL-6 were significantly higher in the OSAS group than in the obese control group. Moreover, after treatment in the OSAS group, significant decreases were observed in the concentration of CRP and IL-6. Another study conducted by Jin and colleagues found increased IL-8, TNF-α, CRP, ICAM-1, VCAM-1, and selectin levels in the OSAS group [63]. After 3 months of CPAP therapy, the inflammatory factors were significantly decreased. Similarly, several studies demonstrated decreased expression levels of ICAM1, IL-8, HMGB1, and SSA were observed in OSAS subjects who followed CPAP treatment [64–66]. Additionally, the study performed by Lu et al. [67] found elevated levels of NF-κB and hypoxia-inducible factor-1α (HIF-1α) in OSAS patients that decreased after CPAP treatment. Furthermore, the low level of surfactant protein D (SPD) in OSAS was increased due to CPAP. Tichanon et al. [68] assessed airway inflammation by measuring the levels of fractional exhaled nitric oxide (FeNO). Decreased levels of FeNO were observed after 3 months of CPAP treatment in OSAS patients compared with pre-CPAP. However, many studies have failed to document any significant effects of CPAP treatment on inflammatory marker levels. The discrepancy with the results above may be due to the short therapy duration, inadequate compliance, and comorbidities such as cardiovascular disease (CVD). Therefore, more randomized controlled trials, especially those with longer treatment duration, adequate compliance are needed to elucidate the real effects of CPAP on inflammatory response in OSAS patients.

**Table 2:** Inflammation levels after CPAP treatment in OSAS patients

Reference	Number of patients	Treatment duration	Daily duration	Detecting parameter	Before treatment	After treatment
Schiza et al. 2010 [60]	528	12 months	≥ 4 h/night	CRP	CRP↑	CRP↓
Steiropoulos et al. 2009 [61]	52	6 months	≥ 4 h/night	TNF-α, IL-6, UA and CD4^+^ cell count	TNF-α↑ UA↑ CD4^+^ cell count↑	TNF-α↓ UA↓ CD4^+^ cell count↓
Yokoe et al. 2003 [62]	30	1 month	NA	CRP, IL-6	CRP↑ IL-6↑	CRP↓ IL-6↓
Jin et al. 2017 [63]	100	3 months	NA	IL-8, TNF-α, CRP, ICAM-1, VCAM-1, and selectin	IL-8↑ TNF-α↑ CRP↑ ICAM-1↑ VCAM-1↑ selectin↑	IL-8↓ TNF-α↓ CRP↓↓ ICAM-1↓ VCAM-1↓ selectin↓
Wu et al. 2010 [64]	30	2 months	NA	HMGB1 and NOx	HMGB1↑ NOx ↓	HMGB1↓ NOx↑
Kuramoto et al. 2009 [65]	116	3 months	NA	SAA, CRP	CRP↑ SSA↑	SAA↓
Ohga et al. 2003 [66]	20	8–18 months	NA	ICAM-1, IL-8, MCP-1	ICAN-1↑ IL-8↑ MCP-1↑	ICAM-1↓ IL-8↓
Lu et al. 2017 [67]	58	2 months	NA	NF-κB, HIF-1α and SPD	NF-κB↑ HIF-1α↑ SPD↓	SPD↑ NF-κB↓ HIF-1α↓
Tichanon et al. 2016 [68]	13	3 months	≥ 5 h/night	FeNO, MDA	FeNO↑ MDA↑	FeNO↓ MDA↓

*CRP* C-reactive protein, *TNF*-*α* tumor necrosis factor-α, *IL* interleukin, *ICAM*-*1* intercellular adhesion molecules-1, *VCAM*-*1* vascular cell adhesion molecule-1, *UA* uric acid, *NF*-*κB* nuclear factor kappa B, *HMGB1* high mobility group box 1, *NOx* nitric oxide derivative, *SSA* serum amyloid A, *MCP*-*1* monocyte chemoattractant protein-1, *HIF*-*1α* hypoxia-inducible factor-1α, *SPD* surfactant protein D, *FeNO* fractional exhaled nitric oxide, *MDA* malondialdehyde, *NA* not administrated

### Mechanisms of Inflammation in OSAS

The increased evidence collected over several years supports that OSAS should be viewed as low-grade chronic inflammatory diseases and the existence of inflammation can be considered a potential contributing factor to OSAS pathophysiology and comorbidity [70]. Numerous studies have established that CIH [71–73], sleep deprivation [74, 75], and snoring [76] are implicated in the activation and progression of inflammation in OSAS patients. A close link exists between hypoxia and inflammation [73]. Previous works have reported that different organs in the hypoxic environment exhibit different responses at the transcriptional, translational, and post-translational levels [77, 78]. HIF-1α, a pivotal transcription factor in hypoxic induction, activates iNOS gene expression, contributing to increased NO synthesis. NO plays a critical role in the initiation and regulation of the inflammatory process [79]. Several studies have demonstrated that hypoxia in OSAS may result in adipose tissue inflammation, leading to insulin resistance [72, 80]. Leptin, a typical biomarker of obesity produced mainly in white adipose tissue, was also increased in OSAS patients [81]. Intermittent hypoxia is a potent stimulator of leptin. The dysregulation of leptin levels promotes oxidative stress and increased production of IL-6 and TNF-α, which are independently induced by OSAS [82]. Effective resolutions of OSAS can lower leptin levels [83]. A systematic review specifically focusing on sleep disturbance and inflammation noted that two inflammatory cytokines, CRP and IL-6, demonstrate a robust association with sleep disturbance [84]. CRP promotes the expression of ICAM and VCAM and induces monocyte-endothelial cell adhesion. CRP upregulates the transcriptional activity of NF-κB, triggering a significant increase in ICAM and VCAM [85]. Similarly, the binding of TNF-α and tumor necrosis factor receptor 1 (TNF-R1) also stimulates NF-κB activity, leading to increased expression of VCAM-1 and MCP-1 in endothelial cells [86]. The above changes will subsequently contribute to monocyte-endothelial cell adhesion, intensifying the inflammatory responses in endothelial cells and resulting in the dysfunction of endothelial cells and atherosclerosis. In addition to the evidence mentioned above, age, smoking, obesity, alcohol abuse, infection, and psychosocial stress may also play pivotal roles in the activation of inflammation in OSAS patients.

## Cognitive impairment in OSAS

Cognition function is the process in which the human brain receives external information, processes it, and then transforms it into an intrinsic psychological activity to acquire knowledge and apply it. It includes psychological processes such as memory, attention, reasoning, language, calculation, executive and visuospatial function, and is an important component of human advanced nervous function [87–89]. Various factors, such as older age, gender, smoking, alcohol consumption, diabetes, obesity, hypertension, metabolic syndrome, atherosclerosis, Down syndrome, hypothyroidism, apolipoprotein E epsilon 4 (APOE ε4) allele, cardiac diseases, stroke, active psychiatric drug, and OSAS have been proven to facilitate the onset and progression of cognitive dysfunction [90]. Neurocognitive impairment of OSAS patients, occurring in both adults and children, has an adverse impact on patients’ quality of life, learning and work efficiency, and health care utilization.

### Brain tissue damage and neurocognitive dysfunction in OSAS

Systematic and meta-analytic reviews provide robust evidence that OSAS plays a critical role in the emergence and development of a broad spectrum of cognitive dysfunctions: attention and vigilance, verbal and visual delayed long-term memory, visuospatial or constructional abilities, and executive function [9, 91, 92]. OSAS in pediatric populations and adults shows a significant association with attention and vigilance deficits [93–95]. Additionally, many studies have suggested that sustained attention deficits in OSAS patients are positively associated with motor vehicle accident risk [96, 97]. The data provided by several studies revealed significant deficits in verbal but not visual memory [98–100]. A meta-analysis specifically focusing on memory problems in OSAS patients demonstrated significant impairments in immediate and delayed verbal and visuospatial memory and immediate visual recall [101]. Mu et al. [102] using the Memory and Executive Screening (MES) to assess cognitive performance found that the immediate recall capacity is the most sensitive item of cognitive dysfunction. Additionally, a recent meta-analysis [103] revealed that various aspects of executive function (shifting, updating, inhibiting, generativity, and fluid reasoning) were impaired in OSAS patients; moreover, they found that these impairments were improved after CPAP treatment.

By utilizing various imaging technologies to detect the changes appearing in the brains of OSAS patients, an increasing number of studies have suggested that OSAS patients with cognitive impairments are associated with wide-spread structural alterations in diverse brain regions, such as gray and white matter, hippocampus, thalamus, cerebral cortex, brain stem, basal ganglion, frontal, temporal, occipital and limbic lobes, superior frontal gyrus, cingulate gyrus, and cerebellum [104–109]. Many studies have found that compromised gray and white matter integrity is associated with slowed information processing, aberrant emotional functioning, and, more importantly, impaired neurocognitive performance, such as memory, attention, and executive function [110, 111]. Moreover, intermittent hypoxia during sleep in OSAS can contribute to apoptosis and atrophy within the structure of the hippocampus, resulting in learning, mnemonic, attentional, and executive function deficits. Macey and colleagues found the hippocampus exhibits sex-specific regional volume increases and decreases in newly diagnosed, untreated OSAS patients [112]. Similarly, Cross et al. [113] evaluated the cortical thickness and subcortical volumes of the brain by magnetic resonance imaging (MRI) in older adults with or without OSAS. The results showed that oxygen desaturation was significantly associated with reduced cortical thickness in both the left and right temporal lobes, leading to reduced verbal encoding. However, sleep disturbance was correlated with increased thickness in the right postcentral gyrus, pericalcarine, and pars opercularis and increased volume of the hippocampus and amygdala. According to the two studies above, a hypothesis is proposed that the increased thickness of cortex and elevated volume of subcortical structures could be interpreted as enlargement or hypertrophy involving reactive or maladaptive mechanisms, such as cerebral edema, neuronal branching, inflammation, glial activation, or even accumulative Aβ deposition. The disease process may result in decreased thickness and volume due to neuronal cell apoptosis and neuronal tissue atrophy. Moreover, this difference in the thickness and volume of brain structures may indicate a distinct time course within which OSAS exerts detrimental effects on brain integrity. Kim and colleagues used MRI images of the brain to assess local volume changes and, identified atrophy of the neocortex and cerebellum and decreased volume of the hippocampal dentate gyrus and cerebellar dentate nucleus in untreated OSAS patients as well as prefrontal atrophy in very severe OSAS patients. Furthermore, they indicated that CPAP treatment is a significant factor correlated with brain structural recovery [114]. Castronovo et al. [115] measured white matter integrity in OSAS patients by diffusion tensor imaging (DTI) and found a decrease in white matter fiber integrity in multiple subdomains of the brain. Moreover, they found that after 12 months of CPAP treatment, both voxel-based morphometry (VBM) and DTI indicated significant improvements in all the affected regions, suggesting that some of the abnormalities are not permanent and can be reversed after effective treatment. Furthermore, the reversibility of the cognitive deficits and corresponding brain morphology changes after treatment have also been confirmed by Canessa et al. [116] using combined neuropsychologic testing and VBM. In the same study, they found a reduced gray matter volume in the left hippocampus (entorhinal cortex), left posterior parietal cortex, and right superior frontal gyrus. After treatment, the increased gray matter volume in the entorhinal cortex, frontal, and parietal structures correlated with improvements in verbal and visuospatial short-term memory, attention, and executive function. Therefore, adherence to treatment such as CPAP and surgery can lead to not only clinical but also brain structural recovery.

### Relationship between cognitive dysfunction and OSAS

Findings from prospective studies of OSAS and cognitive deficits along with results from observational and experimental studies demonstrated that sleep fragmentation and hypoxemia are the two most likely risk factors for cognitive decline [11, 117].

A recent meta-analysis demonstrated that individuals exposed to sleep deprivation showed deficits in attention, memory, and general cognition [9]. A study by Zhang et al. [118] found that OSAS plays a significant and independent role in time- and event-based prospective memory deficits in stroke patients. Their data also suggest that sleep disruption and hypoxemia are the two most important predictors of cognitive dysfunction. Similarly, a link between sleep deprivation and impaired attention and memory maintenance has also been demonstrated by Stepan et al. [119]. Additionally, similar deficits in cognitive function were manifested in children exposed to sleep disruption [120]. Recurrence of the cessation of nocturnal breathing leads to repeat arousal during sleep and subsequent excessive daytime sleepiness, which is one of the hallmark characteristics of OSAS patients. A recent review summarized that sleep disruption and daytime sleepiness mainly influenced attention, vigilance, learning, and memory function, and hypoxia has been proven to be an important predictor of frontal impairment and executive deficits [121]. However, in a prospective controlled study, patients with excessive daytime sleepiness only showed executive dysfunction but no other cognitive impairments [122]. Numerous studies have suggested that OSAS—more specifically, hypoxemia—might cause neuronal damage in multiple regions of the brain, especially in the hippocampus and frontal cortex, leading to attention impairments, slow processing speed, and damaged executive functions [123]. In an IH rat model study, Gao and colleagues utilized Morris water maze tasks to assess the influences of IH exposure on spatial memory and learning performance and found that exposure to IH resulted in poor performance in the tasks above [124]. Additionally, they found that the expression levels of apoptosis and anti-apoptosis proteins both changed in the hippocampus among the IH exposed rats. In another animal study, the expression of brain-derived neurotrophic factor (BDNF) was reduced significantly in mice exposed to CIH and the declined level of BDNF is a crucial factor leading to the impairment of long-term hippocampal plasticity and memory function [125]. Furthermore, besides sleep fragmentation and hypoxemia, numerous studies have demonstrated that age, obesity, hypercapnia, intelligence, and heredity also play an important role in neurocognitive dysfunction [126–128].

## Role of inflammation in the development of cognitive dysfunction in OSAS

Compared with other parts of the body, the brain requires more energy and oxygen consumption and is more sensitive to hypoxia [129]. Data provided by previous studies have generally considered that the activation of inflammation in OSAS patients is a major pathological factor associated with CVD, diabetes mellitus, and nervous system diseases such as dementia, Parkinson’s disease, Alzheimer’s disease, and epilepsy [8, 130, 131]. More importantly, inflammation causes endothelial cell dysfunction and atherosclerosis within the brain, decreasing the brain blood flow and lowering the metabolic function and oxygen consumption in neurons [132]. These changes trigger apoptosis and necrosis of nerve cells and consequently induce various neurocognitive disorders.

### Association of inflammation and cognitive dysfunction in OSAS patients

Because plasma samples are easily available, several studies have measured serum inflammatory biomarker levels and cognitive performance in OSAS patients to observe the association between inflammation and neurocognitive deficits.

The observational study by Huang and colleagues investigated the status of proinflammatory cytokines and cognition in 47 nonobese OSAS children and 32 healthy control children [27]. They respectively examined the plasma levels of inflammatory cytokines such as CRP, TNF-α, IL-1, IL-6, IL-10, IL-17, and IL-23 and investigated neurocognitive functions using neuropsychological tests such as the Wechsler-R intelligence (WPPSI-R) intelligence test to assess IQ score, Conners’ Kiddie Continuous Performance Test (k-CPT) to measure attention problems, and Wisconsin card sorting test (WCST) to evaluate executive function. The standardized regression test indicated a significant relationship between proinflammatory cytokines and neurocognitive performance. The experiment suggested that elevated cytokines such as CRP, THF-α, IL-17, and IL-23 are related to impaired inattention and vigilance abilities. Additionally, the elevated levels of TNF-α, IL-6, and IL-23 were related to decreases in executive functions. Sun et al. [133] used the Montreal Cognitive Assessment (MoCA), Mini-Mental State Examination (MMSE), and Epworth Sleepiness Scale (ESS) to assess the cognitive status in OSAS patients. They found significant impairments in visual space, attention, executive function, and delayed memory function. Elevated levels of hs-CRP, leptin, and TNF-α were observed in the severe OSAS group. More importantly, after adjusting for confounding factors such as BMI, age, and education years, the MoCA scores exhibited negative correlations with AHI, the oxygen desaturation index (ODI), and TNF-α and a positive correlation with minimum oxygen saturation. Notably, after CPAP treatment, both inflammation and cognitive impairment were improved in OSAS patients. In another study conducted by Haensel et al. [134], 39 patients with untreated sleep apnea were recruited. They assessed the concentrations of IL-6, TNF-α, and soluble TNF-R1 (sTNF-R1) and evaluated cognitive domains of attention and working memory, executive function, verbal learning and memory, visual learning and memory, and verbal fluency via a series of psychological tests, such as the Wechsler Adult Intelligence Scale-III Digit Symbol, Symbol Search, Digit Span, and Letter-Number Sequencing; Brief Visuospatial Memory Test Revised; Hopkins Verbal Learning Test Revised; Trail Making A/B; Digit Vigilance Test; Stroop Color-Word Test; and Controlled Oral Word Association Test. Multivariate analyses indicated that only the inflammatory cytokine sTNF-R1 was related to the impaired cognitive function. They also found that sTNF-R1 acted as an important predictor of cognitive status. Taken together, these findings above suggest that the elevated inflammation levels contribute, at least partially, to CIH-mediated neuronal damage and cognitive dysfunction.

### Inflammation leading to cognitive deficits in CIH animals

Due to the limitations of clinical trials, researchers can only screen for cognitive morbidity via different brain imaging studies and various neuropsychological tests. Animal models have provided considerable benefits in various studies, particularly, regarding interventions that are difficult or impossible to perform on humans. Animal experiments provide an ideal solution to these problems. Thus far, CIH mice have been widely used to elucidate the underlying mechanisms of cognitive impairment in OSAS (Table 3).

**Table 3:** The relationship between inflammation and cognitive impairment in OSAS animal model

Reference	Experiment animal	Control animal	Detecting parameter	Cognitive dysfunction	Results
Dong et al. 2018 [135]	V + CIH; SEV + CIH	V + RA; SEV + RA	TNF-α, IL-1β, activity of microglia, and expression and activity of PPAR-γ in hippocampus	Impaired spatial learning and memory in experiment group. SEV exaggerated the cognitive deficits	V + CIH showing increased TNF-α, IL-1β levels and microglia activity. SEV aggravated microglia-mediated inflammation via downregulation of PPAR-γ
Sapin et al. 2015 [136]	C57BL/6J mice + IH	C57BL/6J mice + RA	CCL5, MCP-1/CCL2, ICAM-1, TNF-α, IL-1β, IL-6 and IL-10 mRNA and microglial changes in the dH and vH regions of hippocampus	NA	Experiment group showing increased density and morphological features of microglia priming in dH; IL-1β and RANTES/CCL5 mRNA increased in dH of experiment group
Shi et al. 2018 [137]	C57BL/6J mice + IH; T2DM + IH	C57BL/6J mice + RA; T2DM + RA	Hippocampal neurons apoptosis, microglia activity, HMGB1, NF-κB-p65, TNF-α and IL-1β	Longer escape latency; Reduced numbers of platform crossing and percentage of time spent in the fourth quadrant in Morris water maze of experiment group	All the parameters were significantly increased in experiment group
Snyder et al. 2017 [138]	Adult male rats + CIH	Adult male rats + RA	IL-4, IL-5, IL-6, IL-10, IL-13, TNF-α and IFN-γ protein levels and OS levels in brain tissue	NA	Exposure to CIH increases inflammation and OS levels in brain regions associated with neurodegenerative diseases
Darnall et al. 2017 [139]	Rat pups + CIH	Rat pups + RA	Gro/CXCL1 in plasma; IFN-γ, IL-1β, IL-4, IL-5, IL-6, IL-10, IL-13, KC/GRO and TNF-α in brain tissue and NSE	NA	Increased plasma levels of Gro/CXCL1, cerebellar levels of IFN-γ and IL-1β and NSE in rat pups + CIH
Kim et al. 2013 [140]	ALS + CIH; Wt-CIH	ALS + RA; Wt-RA	NF-κB inhibitor alpha, 4-HNE, anti-GFAP and motor neuron counts	Impaired spatial memory in mice exposed to CIH	ALS + CIH showing poor motor learning and spatial memory, higher levels of OS and inflammation and elevated motor neuron death
Block et al. 2003 [141]	Adult rats + IH	Adult rats + RA	Gene expression of TLR4 and mRNA levels of iNOS, COX-2, TNF-α, IL-1β and IL-6 in microglia	NA	All the parameters showing increase in IH group
Deng et al. 2015 [142]	V + CIH; atorvastatin + CIH	V + RA; atorvastatin + RA	TNF-α, IL-1β, MDA, SOD; expression of TLR4, MyD88 and TRIF mRNA and protein; neuronal cell damage in hippocampus CA1 region	NA	All parameters except SOD were increased in V + CIH mice. V + CIH showing lower SOD level. Atorvastatin attenuated all these changes
Burckhardt et al. 2008 [143]	V + IH; GTP + IH	V + RA; GTP + RA	PGE2, RAGE, the ratio of RAGE/β-actin, GFAP, MDA, and p47phox in brain tissue	GTP attenuated IH-induced spatial learning deficits	All parameters were significantly increased in brain tissue of experiment group. GTP alleviated the IH induced inflammation and OS in the brain
Lam et al. 2015 [144]	V + CIH; LBPs + CIH	V + RA; LBPs + RA	TNF-α, IL-1β, COX-2, NFκB, MDA, antioxidant enzymes (SOD, GPx-1), ER stress and apoptosis in the hippocampus	LBPs reversed CIH-induced spatial memory deficits	V + CIH showing increased levels of TNF-α, IL-1β, COX-2, NFκB, ER stress, OS and neuronal apoptosis in hippocampus. LBPs decreased inflammation and OS levels and improved cognitive deficits
Deng et al. 2015 [145]	V + CIH; BBG + CIH	V + RA; BBG + RA	P2X7R mRNA and protein, NFκB, TNF-α, IL-β, IL-6, IL-18, NOX2, SOD, MDA in the hippocampus	BBG improved spatial learning performance in mice exposed to CIH	All parameters showing highest increases in the hippocampus of V + CIH; BBG alleviated CIH induced inflammation, OS, neural injury and cognition deficits
Yuan et al. 2015 [146]	V + CIH; Telmisartan + CIH	V + RA; Telmisartan + RA	Plasma CRP and IL-6; MDA, NOS, NO and apoptosis in hippocampal CA1 region	NA	All parameters showing highest increases in V + CIH. Telmisartan decreased inflammation and OS levels and hippocampal apoptosis
Row et al. 2004 [147]	PAFR^–/–^ mice + IH; Wt + IH	PAFR^–/–^ mice +RA; Wt +RA	NOS activity, COX-2 and PGE2 in cortical; caspase 3 in cortex and CA1 region of hippocampus	Impaired spatial learning showing in Wt + IH but not PAFR^–/–^ mice + IH	Wt + IH showing the highest levels of all the parameters. PAFR^–/–^ alleviated neuroinflammation and apoptosis in the brain

*V* + *CIH* vehicle + CIH, *Wt*-*CIH* wild-type + CIH, *PAFR*^–/–^*mice* + *IH* platelet-activating factor receptor deficient mice + IH, *NA* not administrated, *CIH* chronic intermittent hypoxia, *RA* room air, *SEV* sevoflurane, *ALS* amyotrophic lateral sclerosis, *GTP* green tea catechin polyphenols, *LBPs Lycium barbarum* polysaccharides, *BBG* Brilliant Blue G, *TNF*-*α* tumor necrosis factor-α, *IL* interleukin, *PPAR*-γ peroxisome proliferators-activated receptor γ, *CCL5* CC motif chemokine ligand 5, *MCP*-1/*CCL2* monocyte chemoattractant protein-1/CC motif chemokine ligand 2, *ICAM*-1 intercellular adhesion molecules-1, *HMGB1* high mobility group box 1, *NF*-*κB* nuclear factor kappa B, *IFN*-γ interferon-γ, *OS* oxidative stress, *GFAP* glial fibrillary acidic protein, *NSE* neuron-specific enolase, *4*-*HNE* 4-hydroxynonenal, *TLR4* toll-like receptor-4, *iNOS* inducible nitric oxide synthase, *NOS* nitric oxide synthase, *COX*-*2* cyclooxygenase-2, *MDA* malondialdehyde, *SOD* superoxide dismutase, *GPx*-*1* glutathione peroxidase-1, *MyD88* myeloid differentiation factor 88, *TRIF* TIR domain-containing adaptor inducing interferon-β, *NOX2* NADPH oxidase 2, *PGE2* prostaglandin E2, *RAGE* receptor for advanced glycation end product, *ER stress* endoplasmic reticulum stress, *P2X7R* P2X7 receptor

Dong et al. [135] found the sevoflurane exaggerated microglia-mediated neuroinflammation and aggravated cognitive deficits in CIH rats via downregulation of PPAR-γ in the hippocampus. After IH exposure and administration of sevoflurane, the animals were subjected to the Morris water maze to assess spatial learning and memory and then the levels of proinflammatory cytokines such as TNF-α, IL-1β, and the activity of microglia in the hippocampus were examined. The results showed that microglia activity and TNF-α and IL-1β levels in the hippocampus were increased in CIH rats, with greater increases in CIH + sevoflurane rats. Notably, CIH + sevoflurane rats showed a much longer escape latency to locate the hidden platform and much less time spent in the goal quadrant than the CIH group. Thus, microglia-mediated neuroinflammation exaggerated by sevoflurane in the hippocampus plays an important role in the pathogenesis of CIH-induced cognitive deficits. Sapin and colleagues determined the levels of several inflammatory cytokines and microglial changes in the hippocampus of mice under normoxia or hypoxia conditions [136]. They found that chronic, but not acute IH exposure, induced significant increases in the density and morphological features of microglia priming. However, increased mRNA levels of inflammatory cytokines such as RANTES/CCL5 and IL-1β were observed in acute but not chronic IH-exposed mice. These changes, including early but transient cytokine alterations and delayed but long-term microglia-mediated inflammation in the hippocampus, may lead to cognitive dysfunction and neurodegeneration in IH mice. Shi et al. [137] and colleagues found that microglia activity, the levels of NF-κB-p65, TNF-α and IL-1β, and hippocampal neuronal apoptosis were significantly increased after IH exposure. This increased neuroinflammation and brain tissue damage in IH-exposed mice might explain the poor performance in the Morris water maze test. Additionally, they found that HMGB1 secreted by activated microglia was significantly elevated in vitro. Furthermore, Snyder et al. [138] removed the brain tissue from both the CIH rodent group and normoxia control group to survey the association between inflammation and early-stage neurodegeneration. The CIH group showed increased inflammation and oxidative stress in brain regions associated with early-stage Alzheimer’s disease and Parkinson’s disease. Therefore, the results indicated that IH-induced inflammation may be a key feature in early-stage neurodegenerative diseases. Their data also suggest that inflammatory profiles are altered in a brain region-specific manner in the central nervous system (CNS).

Inflammatory cytokines, brain injury biomarkers, and brain imaging were observed by Darnall et al. [139] to clarify the outcomes of intermittent hypoxia in rodents. Their study showed that the IH-exposed rat pups exhibited increased plasma levels of Gro/CXCL1 and elevated cerebellar IFN-γ and IL-1β levels compared with the room air control group. In the IH-exposed rat pups, brain imaging showed decreased white matter integrity. Moreover, neuron-specific enolase (NSE), which is released after neuronal death and traumatic brain injury, was higher in the medullas of IH-exposed rats compared with that in controls. These findings provided evidence that hypoxia-mediated inflammation and brain injury might later manifest as executive deficits. Kim et al. [140] reported that CIH might aggravate motor learning and spatial memory deficits by lowering the levels of NF-κB inhibitor alpha (IκBα) and exaggerate oxidative stress in amyotrophic lateral sclerosis (ALS) mice. Similarly, Block et al. [141] dissected the cortex, medulla, and spinal cord tissues from IH rats to determine the mRNA levels of inflammatory cytokine TLR4, which plays an important role in the regulation of inflammation. The results presented that IH treatment increased inflammatory gene expression, including that of iNOS, COX-2, TNF-α, IL-1β, and IL-6, in different brain regions. Additionally, the mRNA levels of TLR4 were significantly upregulated by IH and the increase in TLR4 expression is consistent with the timing of peak inflammatory gene expression, suggesting that TLR4 serves as an essential factor in IH-induced inflammation.

Findings from clinical and animal studies demonstrated that inflammation might play a crucial role in neuronal cell injury and consequent cognitive impairments associated with CIH. Both clinical and animal experiments demonstrate learning and memory deficits. Impaired attention/vigilance and executive function were only found in clinical studies. Elevated plasma and brain tissue CRP, IL-1β, IL-6, TNF-α, HMGB1, NF-κB, TLR4, and COX-2 levels were observed in CIH animal models. These inflammatory cytokine alterations in animal studies are consistent with the changes in human plasma. Additionally, animal experiments demonstrated increased microglial activation and neuronal apoptosis in various regions of the brain. CPAP has been proven to alleviate CIH-mediated inflammation and cognitive dysfunction in clinical studies.

### Potential mechanisms between inflammation and neurocognitive dysfunction

**Fig. 1:**
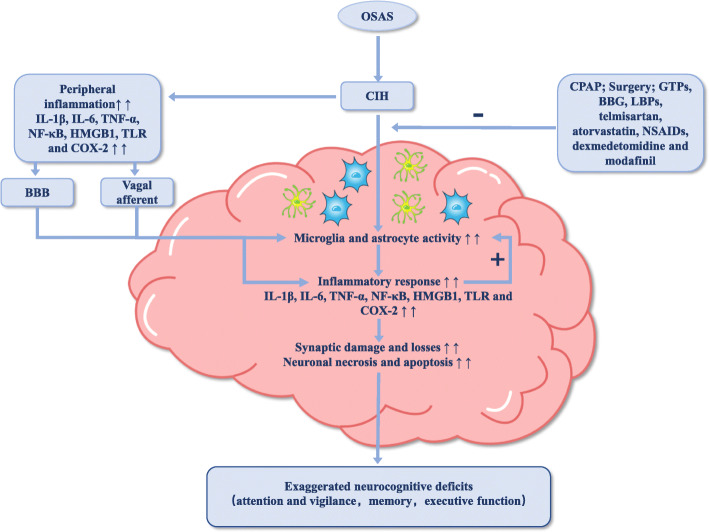
Inflammation and cognitive deficits in OSAS. This figure demonstrates the important role played by inflammation in OSAS related cognitive dysfunction. CIH characterized in OSAS leads to peripheral inflammation which access the CNS through BBB or via the stimulation of vagal afferents. The high level of inflammation in the CNS further upregulates glial cells (microglia and astrocyte) activity, inducing and aggravating the neuroinflammatory reaction. Meanwhile, CIH could directly activate microglia and astrocyte, prompting the release of inflammatory cytokines in the CNS. The excessive neuroinflammatory response could in turn boost the activation of glial cells, lead to synaptic damage and losses, neuronal necrosis and apoptosis, and ultimately result in exaggerated neurocognitive deficits. In addition, treatment with CPAP, surgery, and administration of GTPs, BBG, LBPs, telmisartan, atorvastatin, NSAIDs, dexmedetomidine, and modafinil would alleviate the neuroinflammation and improve cognitive function. *CIH* chronic intermittent hypoxia, *CNS* central nervous system, *BBB* blood-brain barrier, *CPAP* continuous positive airway pressure, *GTPs* green tea catechin polyphenols, *BBG* Brilliant Blue G, *LBPs Lycium barbarum* polysaccharides, *IL* interleukin, *TNF*-*α* tumor necrosis factor-α, *NF*-*κB* nuclear factor kappa B, *COX*-*2* cyclooxygenase-2, *NSAIDs* non-steroidal anti-inflammatory drugs

As discussed previously, OSAS can lead to peripheral and neural inflammation. The relationship between an excessive inflammatory response and impaired cognitive function has been documented in many other diseases, such as sepsis, Alzheimer’s disease, post-operative cognitive dysfunction (POCD), traumatic brain injury, and spinal cord injury [148–151]. Based on the existing evidence, the relationship between inflammation and cognitive dysfunction in OSAS can be inferred as shown in Fig. 1.

The blood-brain barrier (BBB) is a physical and biochemical barrier that isolates the CNS from the peripheral environment. It plays an important role to maintain cerebral homeostasis [152]. Due to the protective effect of BBB, the CNS was previously thought to be impervious to peripheral inflammation. However, the recent discovery of the bidirectional cross-talk between peripheral and neural inflammation has challenged this assumption [153]. Cumulative evidence has suggested that peripheral inflammation is a major factor that can lead to neuroinflammation by damaging endothelial cells, disrupting the integrity and permeability of the BBB [154]. Many studies have documented that the release of TNF-α and downstream NF-κB can breach the BBB, facilitate the migration of macrophages into the hippocampus, activate glial cells, and eventually lead to cognitive deficits after peripheral surgery [155, 156]. The elevated serum levels of HMGB1 in sepsis may also act on cerebral microvascular endothelial cells, compromise the integrity of BBB, expose the brain to neurotoxic substances, and finally contribute to cognitive dysfunction [151]. Additionally, the BBB can selectively transport several inflammatory cytokines such as IL-1β, IL-6, and TNF-α—via specific receptors and transporters in cerebral endothelial cells [157]. Additionally, inflammatory cytokines can also access the brain through the circumventricular region where the BBB is discontinuous [158]. Notably, some studies have found that peripheral cytokines directly stimulate the vagal afferents to convey the peripheral immune signals to the CNS [159].

Glial cells are another large category of cells in the CNS besides neurons. Microglia, as resident immune cells of the CNS, play an important role in the regulation of inflammation in the brain. Neuroinflammation is specifically manifested by glial cell activation. CIH and the invasion of peripheral inflammatory factors activate microglia and astrocytes, which secrete cytokines, such as IL-1β, IL-6, TNF-α, and HMGB1, oxidative species, adhesion molecules, and other signalling mediators [160]. Many studies have documented that the high levels of cytokines produced by microglia and astrocytes can aggravate neuronal axon and synaptic damage, increase demyelination, and impair the integrity of white matter in multiple CNS regions [160, 161]. The cytokines released by glial cells can further disrupt the BBB, and activate glial cells, leading to a vicious cycle. Evidence has shown that the inflammatory responses in an animal model of surgery result in decreased levels of BDNF, which is crucial in multiple aspects of neural plasticity [125, 162]. Hippocampus, which is implicated in learning and memory function, appears particularly vulnerable to excessive neuroinflammation due to abundant receptors such as IL-1β receptor, TNF-α receptor, and HMGB1 receptor in this region [163]. These researches gave us a much more distinct view on the relationship between the inflammatory status and the potential relationships with specific cognitive functions. However, the mechanism between inflammation and cognitive decline has been rarely mentioned in OSAS and was mainly discussed on other diseases, such as Alzheimer’s disease, POCD, and sepsis. Therefore, further clinical and animal experiments are required to clarify the precise mechanism and specific signaling pathways involved in the pathogenesis of OSAS-mediated brain structural damages and cognitive alteration.

### Potential therapies for inflammation-mediated neurocognitive dysfunction

Numerous studies have examined various endogenous factors and exogenous substances that would protect brain tissues from CIH-induced neuroinflammatory impairment. Many anti-inflammation drugs and substances can effectively suppress the inflammatory process and improve cognitive dysfunction. Intracerebroventricular injection of anti-HMGB1 antibody can inhibit the synthesis of inflammatory cytokines, microglia activation, and neuronal damage in the hippocampus after status epilepticus [164]. Terrando et al. demonstrated that the blockade of TNF-α leads to lower levels of downstream IL-1, alleviates neuroinflammation, and improves neurocognition in an animal model of surgery-induced cognitive decline [165]. Similarly, in a rat experiment, intracisternal administration of IL-1 receptor antagonist inhibited neuroinflammation and cognitive decline after surgery [166]. TLR, a critical immune receptor that binds specifically to pathogen-associated molecular patterns (PAMPs) to mediate inflammatory reaction, is associated with almost all inflammation-related diseases. Administration of a TLR-4 receptor antagonist also plays a role in improving neuroinflammation and cognition in an Alzheimer’s disease mouse model [150]. Nonsteroidal anti-inflammatory drugs (NSAIDs) block the synthesis of prostaglandins by inhibiting cyclooxygenase enzymes (COX-1 and COX-2), thereby suppressing inflammatory activities [167]. Numerous studies have found that high levels of cytokine expression (IL-1β, IL-6, and TNF-α), glial activation, hippocampal microgliosis, and cognitive deficits are ameliorated by the administration of NSAIDS such as ibuprofen, paracetamol, and parecoxib in animal models [168–170].

In addition to the anti-inflammatory drugs mentioned above, dexmedetomidine, an α2 adrenoceptor agonist, can lower the levels of IL-1β, IL-6, TNF-α, and TLR-4 in the hippocampus, reduce the activity of glial cells, and reverse neurodegenerative and neuronal apoptosis, thus improving cognitive function [171, 172]. Deng et al. [142] found that atorvastatin, which regulates the expression of TLR4, attenuated neuronal damage and decreased the elevation of TLR4 together with downstream inflammatory cytokines such as TNF-α and IL-1β in the hippocampus. Green tea catechins polyphenols (GTPs) extracted from green tea could alleviate IH-induced spatial learning deficits. Moreover, the GTPs also decreased the high levels of inflammation and OS in IH-exposed animals [143]. Similarly, Lam et al. [144] demonstrated that *Lycium barbarum* polysaccharides (LBPs) play a neuroprotective role in IH-induced, cognition-impaired rats. Their study showed that LBPs significantly lower the high levels of inflammation and OS in subfields of the hippocampus, prevent autophagic flux and apoptosis induced by hypoxia, and attenuate IH-mediated spatial memory deficits in IH rat models. Brilliant Blue G, a selective P2X7R antagonist, was confirmed to prevent IH-induced neuronal cell apoptosis and spatial learning deficits by inhibiting inflammation and oxidative stress in the hippocampus of CIH murine models [145]. Telmisartan, an angiotensin II type 1 receptor blocker, exerts protective effects on hippocampus damage induced by CIH. Yuan and colleagues utilized TUNEL staining for apoptotic cells to evaluate hippocampal injury [146]. They found that treatment with telmisartan effectively ameliorates CIH-induced hippocampal apoptosis. Moreover, their data demonstrated that treatment with telmisartan suppressed the elevated levels of inflammation and OS in the peripheral blood and hippocampus of CIH rat models. This finding suggests that the neuroprotective effects of telmisartan may be mainly due to the inhibition of inflammation and oxidative stress levels. Platelet-activating factor (PAF), an endogenous proinflammatory phospholipid, is synthesized in the CNS [173]. Row et al. [147] found that IH-exposed PAF receptor-deficient mice showed lower inflammation levels, better neurocognitive function, and less neuronal apoptosis than wild-type IH mice. These findings demonstrate that IH-induced neuron injury and cognitive deficits are mediated by PAF receptor via the activation of COX-2 and iNOS in the brain tissue. Notably, modafinil, a psychostimulant drug mainly used in the treatment of sleep disorders, was recently found to have neuroprotective effects. Modafinil can alleviate neural and systemic inflammation by inhibiting the activation of microglial and infiltration of leukocytes into the CNS, thereby reversing neurological disorders in animal models [174]. These neuroprotective substances may be a promising therapeutic method to alleviate OSAS-mediated cognitive impairments and more studies are needed in the future to verify this link.

## Conclusion

Repeated courses of airway collapse and obstruction in OSAS patients induced recurrent apnea and periodic arousal during sleep, leading to IH and EDS and contributing to the occurrence and development of neuroinflammation and consequent neurocognitive impairments. Because the hippocampus and cortex are most sensitive to hypoxia, most neurocognitive dysfunctions are related to these two regions. Inflammatory cytokines including IL-1β, IL-6, TNF-α, NF-κB, HMGB1, and COX-2 are involved in the development of neurocognitive deficits in OSAS patients. Treatment with CPAP, atorvastatin, telmisartan, GTPs, Brilliant Blue G, LBPs, and anti-inflammatory substances can attenuate the IH-induced neuroinflammation, leading to better neurocognitive function. These findings suggest an intimate link between inflammation and cognitive impairment in OSAS and provide a novel direction for the treatment of related disorders in the future.

## Data Availability

The data supporting the conclusion of this article is included within the “References” section.

## Abbreviations

OSAS: Obstructive sleep apnea syndrome
CIH: Chronic intermittent hypoxia
CPAP: Continuous positive airway pressure
EDS: Excessive daytime sleepiness
IL: Interleukin
hs-CRP: High-sensitivity C-reactive protein
TNF-α: Tumor necrosis factor-α
NF-κB: Nuclear factor kappa B
ICAM: Intercellular adhesion molecules
VCAM: Vascular cell adhesion molecule
VEGF: Vascular endothelial growth factor
MCP-1: Monocyte chemoattractant protein-1
MIF: Macrophage migration inhibitory factor
CCL5: CC motif chemokine ligand 5
COX-2: Cyclooxygenase-2
MRP 8/14: Myeloid-related protein 8/14
P2X7R: P2X7 receptor
IFN-γ: Interferon-γ
HMGB1: High mobility group box 1
SSA: Serum amyloid A
PPAR-γ: Peroxisome proliferators-activated receptor γ
PTX-3: Pentraxin-3
PGE2: Prostaglandin E2
UA: Uric acid
NO: Nitric oxide
iNOS: Inducible nitric oxide synthase
TLRs: Toll-like receptors
RAGE: Receptor for advanced glycation end product
BMI: Body mass index
AHI: Apnea hypopnea index
ESR: Erythrocyte sedimentation rate
TST90: Sleep time spent with SaO_2_ less than 90%
Aβ: Amyloid beta
ProCT: Procalcitonin
HIF-1α: Hypoxia inducible factor-1α
FeNO: Fractional exhaled nitric oxide
CVD: Cardiovascular disease
TNF-R1: Tumor necrosis factor receptor 1
APOE ε4: Apolipoprotein E epsilon 4
MRI: Magnetic resonance imaging
DTI: Diffusion tensor imaging
VBM: Voxel-based morphometry
BDNF: Brain-derived neurotrophic factor
ODI: Oxygen desaturation index
CNS: Central nervous system
NSE: Neuron-specific enolase
IκBα: NF-κB inhibitor alpha
ALS: Amyotrophic lateral sclerosis
POCD: Post-operative cognitive dysfunction
BBB: Blood-brain barrier
PAMPs: Pathogen-associated molecular patterns
NSAIDs: Nonsteroidal anti-inflammatory drugs
GTPs: Green tea catechins polyphenols
LBPs: *Lycium barbarum* polysaccharides
PAF: Platelet-activating factor
MDA: Malondialdehyde
OS: Oxidative stress
SOD: Superoxide dismutase
NOX_2_: NADPH oxidase 2
4-HNE: 4-Hydroxynonenal
TRIF: TIR domain-containing adaptor inducing interferon-β
GPx-1: Glutathione peroxidase-1
MyD88: Myeloid differentiation factor 88
